# Physical Activity Modifies the Severity of COVID-19 in Hospitalized Patients—Observational Study

**DOI:** 10.3390/jcm12124046

**Published:** 2023-06-14

**Authors:** Edyta Sutkowska, Agata Stanek, Katarzyna Madziarska, Grzegorz K. Jakubiak, Janusz Sokołowski, Marcin Madziarski, Karolina Sutkowska-Stępień, Karolina Biernat, Justyna Mazurek, Adrianna Borowkow-Bulek, Jakub Czyżewski, Gabriela Wilk, Arkadiusz Jagasyk, Dominik Marciniak

**Affiliations:** 1 University Rehabilitation Centre, Wroclaw Medical University, 50-556 Wroclaw, Poland; 2 Department and Clinic of Internal Medicine, Angiology, and Physical Medicine, Faculty of Medical Sciences in Zabrze, Medical University of Silesia, 41-902 Bytom, Poland; astanek@tlen.pl (A.S.); grzegorz.k.jakubiak@gmail.com (G.K.J.); 3 Clinical Department of Nephrology and Transplantation Medicine, Wroclaw Medical University, 50-556 Wroclaw, Poland; katarzyna.madziarska@umw.edu.pl; 4 Clinical Department of Emergency Medicine, Wroclaw Medical University, 50-556 Wroclaw, Poland; 5 Clinical Department of Rheumatology and Internal Medicine, University Hospital, 50-556 Wroclaw, Poland; mmadziarski@usk.wroc.pl; 6 Department of General, Minimally Invasive and Endocrine Surgery, University Hospital, 50-556 Wroclaw, Poland; ksutkowska@usk.wroc.pl; 7 Department of Internal Medicine, Angiology and Physical Medicine, Specialist Hospital No.2, 41-902 Bytom, Poland; adrianna.borowkow@gmail.com; 8 Postgraduate–Internship, University Hospital, 50-556 Wrocław, Poland; 9 Department of Drugs Form Technology, Wroclaw Medical University, 50-556 Wroclaw, Poland

**Keywords:** COVID-19, IPAQ, disease severity, physical activity level, infection

## Abstract

Background and aim: Physical activity (PA) can modulate the immune response, but its impact on infectious disease severity is unknown. We assess if the PA level impacts the severity of COVID-19. Methods: Prospective, cohort study for adults hospitalized due to COVID-19, who filled out the International Physical Activity Questionnaire (IPAQ). Disease severity was expressed as death, transfer to intensive care unit (ICU), oxygen therapy (OxTh), hospitalization length, complications, C-reactive protein, and procalcitonin level. Results: Out of 326 individuals, 131 (57; 43.51% women) were analyzed: age: median—70; range: 20–95; BMI: mean—27.18 kg/m²; and SD: ±4.77. During hospitalization: 117 (83.31%) individuals recovered, nine (6.87%) were transferred to ICU, five (3.82%) died, and 83 (63.36%) needed OxTh. The median for the hospital stay was 11 (range: 3–49) for discharged patients, and mean hospitalization length was 14 (SD: ±5.8312) for deaths and 14.22 days (SD: ±6.92) for ICU-transferred patients. The median for MET-min/week was 660 (range: 0–19,200). Sufficient or high PA was found in recovered patients but insufficient PA was observed in dead or ICU-transferred patients (*p* = 0.03). The individuals with poor PA had a higher risk of death (HR = 2.63; ±95%CI 0.58–11.93; *p* = 0.037). OxTh was used more often in the less active individuals (*p* = 0.03). The principal component analysis confirmed a relationship between insufficient PA and an unfavorable course of the disease. Conclusion: A higher level of PA is associated with a milder course of COVID-19.

## 1. Introduction

The course of coronavirus disease 2019 (COVID-19) was often surprising and sometimes independent of variables previously considered poor prognostic factors [1]. One of the unfavorable prognosis factors was age; however, diabetes and, most of all, obesity were also detected [2,3,4]. These factors have a common denominator: low physical activity (PA). However, healthy young people also show different levels of PA [5,6]. Thus, also in this group, different disease courses could be observed [7]. This directs our attention to the possible relationship between the course of the disease and PA.

There are a lot of data confirming the impact of physical exercise on human health. Posadzki et al. published in 2020 the systematic Cochrane review [8] which analyzed data from 27,671 participants in the context of the effects of physical activity on health outcomes. One of the most important conclusions was that individuals can significantly modify their physical and mental health by exercising more. The reduction of mortality was also reported, mainly in studies with a longer follow-up, and can reach up to 35% when comparing active people with those characterized by a sedentary lifestyle [9]. Physical activity also improves mental well-being and quality of life (QoL), the very important factors of human life that are involved in the physical status of individuals [10,11].

The balance between the infection and innate as well as adaptive immune response seems to play a crucial role in the course of the infectious disease. The severe acute respiratory syndrome coronavirus-2 (SARS-CoV-2) can effectively evade early innate immune responses, and thus multiply more efficiently before the most effective adaptive immune response is ready. In young, lean, healthy, and active individuals, the first step in the immune response works more effectively and adequately mobilizes T cells from the adaptive immune system to reduce the viral load. In the individuals characterized by a dysfunctional T-cell population (e.g., older, obese people), excessive inflammation with tissue damage is observed as a “response” to diminished adaptive immunity [12,13,14]. Thus, the impairment in one immune response is compensated by the other. Physical activity can modulate the level of inflammation in pathological states [15,16,17,18] so it is also possible that it can modify the cytokine storm, the main cause behind many organ failures and, finally, patient death during severe acute respiratory syndrome coronavirus-2 infection [19,20]. The proposed mechanisms of action of PA in the course of COVID-19 involve irisin production, AMPK pathway activation [21], macrophage activity regulation, and many others [20,22]. The wide aspect of how PA can keep the immune system in a good condition (including immunometabolism) was excellently described in D.C. Nieman and B.D. Pence’s review [23]. Regular PA can also provide many “macro“ benefits (not only those connected with immune system), such as better cardiovascular and lung health [24,25], which is affected primarily during infection.

Still, their clinical effect on the severity of a disease such as COVID-19 was not part of a prospective study. The main problem which can be detected is the difficulty in assessing the level of PA based on time-consuming questionnaires. The International Physical Activity Questionnaire (IPAQ) is a useful tool for measuring health-related PA in populations [26], and also in its short form (SF). The pandemic provided opportunities to investigate expected relationships between daily PA and disease severity. However, patients hospitalized due to COVID-19 varied in health status, not always having the strength to fill out a questionnaire. In addition, healthcare professionals’ PPE clothing (special masks and suits) significantly limited their ability to interview patients. All of these probably made it difficult to assess the above dependencies for healthcare professionals. 

### Aim

This prospective cohort study aims to investigate whether the level of physical activity measured with the IPAQ-SF modifies the severity of COVID-19. Taking into account the above mention reports, we hypothesized that people who are more physically active have a milder course of the infection.

## 2. Materials and Methods

All the patients were asked to give their written consent to participate in the study. Patient data and results were analyzed anonymously. The Bioethics Committee of the Wroclaw Medical University approval number is: KB 1081/2021. Trial Registration number: NCT05200767.

### 2.1. Place

The study was conducted in two hospitals dedicated temporarily to COVID-19 patients: one in Wroclaw, Lower Silesia, and the other in Bytom, Upper Silesia. Initially, the Wroclaw hospital participated in the project, and information from this hospital was collected from 3 January 2022 to March 2022. After obtaining the required approvals, the Bytom hospital joined the project, and data from this hospital covered the period from 31 January to 11 February 2022. After the mentioned periods, both hospitals were no longer COVID-19 treatment centers.

### 2.2. Participants

Adults (aged 18 at the least) of any sex and race hospitalized for SARS-CoV-2 infection (confirmed by the polymerase chain reaction (PCR) method or equivalent diagnostic technique) at COVID-19 units were asked to give written consent to participate. The exclusion criteria were as follows:

- pregnancy;
- inability to complete the questionnaire during hospitalization or up to a week after discharge;
- history of a significant cardiovascular event in the last 6 months (acute coronary syndrome, stroke, amputation, revascularization of peripheral vessels, and pulmonary or peripheral embolism of any etiology) if the patient did not complete the rehabilitation process;
- symptomatic chronic respiratory disease not responding to therapy before hospitalization for COVID-19 (or no therapy);
- any dyspnea at rest in the last month before COVID-19;
- injury to the locomotor system in the last month before contracting COVID-19;
- hospitalization in the last month before contracting COVID-19.

We proposed these exclusion criteria believing that they could influence the patient’s recent PA level and thus not properly reflect its impact on the course of COVID-19. On the contrary, some conditions, such as a cardiovascular incident or stable chronic pulmonary or locomotor system disease, were accepted for evaluation and did not constitute the exclusion criteria. This is because the patient can fully or almost fully recover after rehabilitation or compensate for disability with some form of activity (e.g., wheelchair use after amputation). In addition, because rehabilitation is time-consuming, we tried to account for it with the proposed periods (e.g., 6 months for cardiovascular incidents).

### 2.3. Methods

The patients were asked to complete the IPAQ during their hospital stay or up to 7 days after discharge. If they were unable to fill out the questionnaire themselves, the patient’s next of kin could also do it on their behalf.

IPAQ expresses PA in metabolic equivalent of task (MET)-min/week units. Based on it, the respondents were assigned one of three categories of activity: insufficient (less than 600), sufficient (600–1500 or 600–3000), or high (more than 1500 or 3000 MET-min/week) [27,28]. Example of IPAQ interpretation: two criteria for high activity: a) vigorous-intensity activity on at least 3 days achieving a minimum of at least 1500 MET-min/week OR b) 7 or more days of any combination of walking, moderate-intensity, or vigorous-intensity activities achieving a minimum of at least 3000 MET-min/week.

Due to the expected poor condition of some patients, the short form of the questionnaire was used. It consists of 7 items on all types of PA related to everyday life, work, and leisure. According to the suggestion of adaptation of the IPAQ for the Polish population (IPAQ-PL), the interviewer helped complete the form [27]. 

The primary outcome measures were death, worsening of the disease (transfer to ICU), or recovery (discharge home). The secondary outcome measure was hospitalization length.

Information on complications, comorbidities, chronic medications, and basic parameters concerning the patient’s status and the disease course was obtained from hospital databases. The following parameters determined the severity of the disease (critical data): death or transfer to ICU, need for oxygen therapy, length of hospitalization, complications other than respiratory failure, and C-reactive protein (CRP) and procalcitonin (PCT) levels on admission. 

The funding sources (Wroclaw Medical University-WMU and Medical University of Silesia-MUS, Wroclaw, Poland) had no impact on the study design, data collection, analysis and interpretation; they also had no role in the writing of the manuscript, but agreed to submit the manuscript for publication and gave financial support for proofreading of the translation (WMU) and article processing charge (both universities). The corresponding author had full access to all of the data and the final responsibility to submit for publication.

### 2.4. Statistical Analysis

Both nominal and ratio variables were analyzed. Basic descriptive statistics were calculated for ratio scale variables: size, mean value—SR, median, 95% confidence interval for the mean value (SR ± 95%CI), and range. Next, size and percentage information was tabulated for nominal variables (including dichotomous ones). 

The normality of data distribution was tested with a Shapiro–Wilk test. We used means and standard deviations (SDs) for normally distributed data and medians and ranges for not normally distributed data.

Relationships between variables of nominal scales (including dichotomous ones) were assessed using Pearson’s chi-squared test.

Pearson correlation coefficient matrices were calculated to determine correlations between variables of ratio scales. Their statistical significance was determined with a *t*-test.

Principal component analysis (PCA) was used to assess global relationships between the key variables analyzed regardless of scale. The developed PCA model was estimated using the NIPALS algorithm. The convergence criterion was set at 0.00001, and the maximum number of iterations was 100. The number of components was determined by establishing the maximum predictive ability of Q^2^ using V-fold cross-validation, adopting $V_{max}=7$. The resulting optimal PCA model was reduced to 2 principal components (PC 1 and PC 2). The results are presented in a graph, including the contribution of each component to the overall percentage of explained variance and information on their statistical significance. 

In addition, survival analysis was performed based on a nonparametric Cox proportional hazards model. 

A significance level of α = 0·05 was assumed for all statistical analyses carried out. 

Statistical analysis was performed using StatSoft’s Statistica 13·3 PL.

## 3. Results

### 3.1. Basic Information

In the mentioned periods, 326 patients (294 in Wroclaw and 32 in Bytom) were hospitalized due to COVID-19, and 140 gave their written consent to participate in the study (124 from Wroclaw and 16 from Bytom) (Supplements Figure S1). However, the final analysis covered 131 cases (57; 43.51% women) because three patients did not fill out the questionnaire, and hospital data on six participants proved insufficient for analysis. The patient age was: median—70; range: 20–95 (N = 131), mean BMI was 27.18 kg/m² (SD: ±4.77; data available for 97 cases); 18 (13.74%) patients were smokers (data available for 125 patients) and 53 (42.06%) were fully vaccinated (data available for 126 individuals) with at least two doses of the vaccine.

A broad characterization of the population is included in the additional documents. They contain information about comorbidities (Supplements Table S1), chronic medications (Supplements Table S2), and laboratory results (Supplements Table S3). Not all of these data were available for the whole group, and the number of analyzed cases is indicated in the tables.

The analysis of the course of hospitalization showed that 117 (83.31%) individuals recovered and were discharged home, nine (6.87%) were transferred to the ICU—their fate is unknown to the researchers, and five (3.82%) died. In 83 (63.36%) individuals, some form of OxTh was necessary. The median for the hospital stay was 11 (range: 3–49) for discharged patients, and mean hospitalization length was 14 days (SD: ±5.8312) for deaths and 14.22 days (SD: ±6.92) for patients transferred to ICU.

### 3.2. Relationship between IPAQ Score and Severity of the Disease

To analyze the relationship between IPAQ score and survival, the group was divided into two subgroups: recovered patients and patients with unfavorable hospitalization outcomes (death or transfer to ICU; N = 14; 10.69%). None of the patients used the help of a family member to complete the questionnaire, but the interviewers helped them to understand it and marked the correct information for all of them to some extent. The median for energy expenditure was 660 MET-min/week (range: 0–19,200), representing the sufficient level of PA regarding the IPAQ. The results of IPAQ are in Table 1.

**Figure 1:**
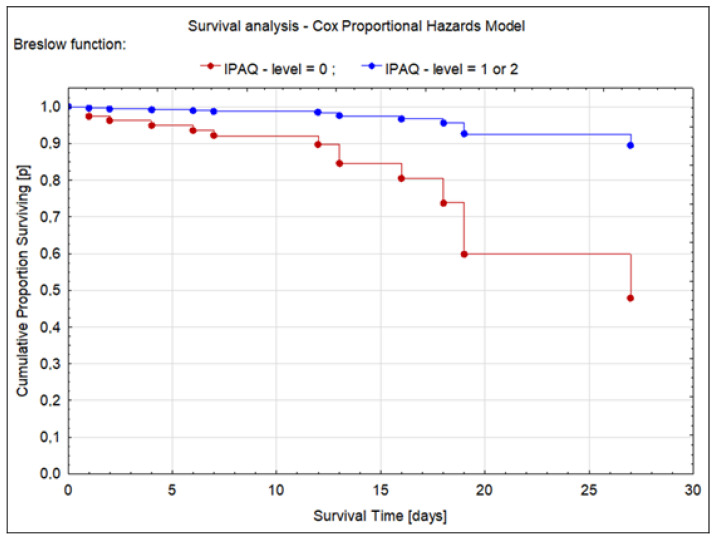
Survival analysis for patients whose physical activity was insufficient (IPAQ = 0) or at least sufficient (IPAQ = 1 or 2).

Sufficient or high PA was found in patients who recovered, while insufficient activity was more common in patients with unfavorable hospitalization outcomes (death or transfer to ICU) (*p* = 0.03) (Table 1). In addition, the individuals with insufficient PA (IPAQ = 0) had a higher risk of death (HR = 2.63; ±95%CI 0.58–11.93; *p* = 0.037), while patients with sufficient or high PA (IPAQ = 1 or 2) were more likely to recover (HR = 0.39; ±95%CI 0.04–4.39; *p* = 0.19). Survival analysis for the participants is presented in Figure 1.

Oxygen therapy was used more often in the less active individuals (IPAQ = 0 and 1) compared to the more active ones (IPAQ = 2) (*p* = 0.03) (Table 1).

There was no difference in hospitalization length between IPAQ = 0 patients (insufficient activity) (median: 12; range: 5–49) and individuals reporting sufficient or high PA (IPAQ = 1 or 2) (median: 11; range: 3–30) (*p* = 0.35).

In 30 (22.90%) individuals, complications other than respiratory failure were observed: for IPAQ = 0, N= 6 for IPAQ = 1 or 2, N = 26. There was no difference between groups regarding the development of complications (*p* = 0.94) and IPAQ score.

The values for CRP and PCT are in Supplements (Table S3). We observed a negative correlation between PA and CRP level on admission (the higher the PA, the lower the CRP, but with no statistical significance) and a positive correlation for PCT level on admission (the lower the PA, the lower the PCT, but also with no significance).

**Figure 2:**
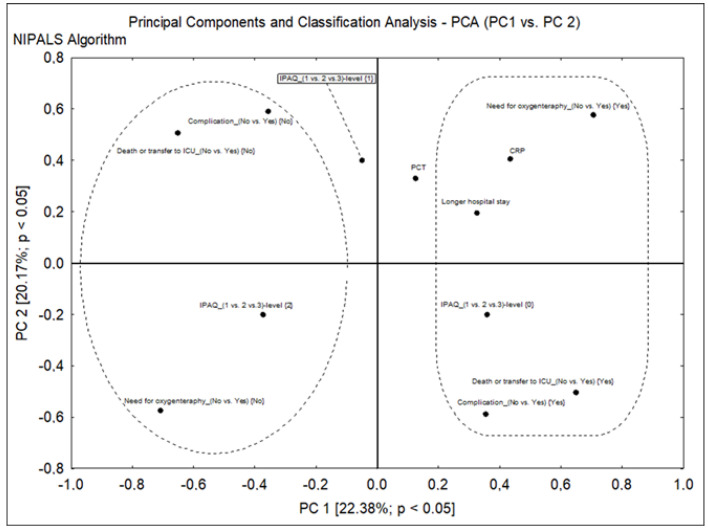
Relationship between physical activity level and selected variables: need for oxygen therapy, complications, duration of hospitalization, and unfavorable hospitalization outcomes (death or transfer to ICU).

The PCA (Figure 2) orders the variables according to how close they are together and thus informs about their relationship. The analysis confirmed a relationship between high PA and no need for OxTh. It also corroborated the relationship between insufficient PA and complications, the need for oxygen therapy, a longer hospital stay, unfavorable hospitalization outcome (death or transfer to ICU), and low PCT but higher CRP on admission.

## 4. Discussion

The exercises build muscle mass and produce benefits such as better glucose control or lipid metabolism [29]. Even though the two are connected with a longer life and better life quality, people, especially younger ones, find it difficult to focus on the long-term benefits and take up regular PA. Thus, information on short-term benefits, such as lower incidence of various infections or their milder course, could encourage it in people of all ages. In addition, the much more severe consequences of COVID-19 (compared to other, more trivial respiratory system infectious diseases) and the significantly higher frequency of serious complications (including high death rate) may also reveal potential protective factors. Therefore, the disclosure of factors that help improve the prognosis could more strongly appeal to the public imagination and mobilize people to take action to improve the course of infection or the occurrence of these diseases in general.

Our study confirms with no doubt that people hospitalized due to COVID-19 who had regular PA before getting sick had a better prognosis, with an about 60% higher chance of recovery, for example, due to a reduced hyper-inflammation response [22], as we observed the relationship between PA level and the main marker of inflammation, CRP (higher PA was characterized by lower CRP), but with no statistical significance. In recent decades, a role for muscle as an endocrine organ producing myokines that can contribute to cross-talk between different organs and thus modify the body’s response to inflammation has been proposed [30]. Therefore, even minimal PA (referred to as sufficient in IPAQ) protects against death or ICU transfer in our study. This level means one of the following: (a) 3 or more days of vigorous activity for at least 20 min per day, OR (b) 5 or more days of moderate-intensity activity or walking for at least 30 min per day, OR (c) 5 or more days of any combination of walking, moderate-intensity, or vigorous-intensity activities achieving a minimum of at least 600 MET-min/week [31].

It is known that PA improves lung function [32]. We also confirmed that being more active reduces the chances of additional procedures such as oxygen therapy. This gives a smoother disease course and indirectly also proves the efficiency of the patient’s respiratory system. Low PA was found by our team to be associated with adverse prognostic factors that appeared during the course of COVID-19, such as the need for oxygen therapy, complications, and, consequently, longer hospital stay, as well as unfavorable hospitalization outcomes (death or transfer to ICU) also in PCA. Thus, our study confirms that, for every person, regardless of fitness and ability, being more active reduces the risk of the adverse, acute consequences of COVID-19 disease. Although we have no information on the long-term complications of COVID-19 up to now, avoiding first-line ones seems to be enough reason to explain to patients the need for minimal but regular PA at least.

In the previous study, we presented patients with type 2 diabetes who declared lower PA levels (mean: 1198•1 MET-min/week) compared to the result of this study, where we focused on the general population [33]. Nonetheless, we are aware that patients with more comorbidities may be less active due to disability [34,35]. The IPAQ analysis combines not only the dynamics of activity but also its regularity and duration as mentioned above. Patients can also pursue a less strenuous PA, such as regular walks, to obtain a higher IPAQ level and reap its benefits. Exercising was found to modulate the AMP-activated protein kinase (AMPK) pathway and decrease cytokine production depending on its intensity, duration, and status [21]. Interestingly, a high-intensity approach is not preferred as it can even be harmful [24,36].

The mean BMI of the studied population was 27 kg/m², and less than 20% of the individuals had BMI ≥ 30 kg/m². As was found in previous studies, obesity was an independent predictor for severity and death in patients suffering from COVID-19 and other infectious diseases [37]. The two hospitals covered in our study were not dedicated to treating the most severe cases; therefore, we cannot exclude that the BMI of the ICU patients was higher [38] as obese individuals are more severely ill and more likely to need mechanical ventilation [39]. Thus, we suggest that the results of our study may be representative of a non-obese population. Due to the independent influence of high BMI mentioned above in the literature on the severity of infection, the analysis of PA’s role on the disease course in the obese population should be an element of a separate investigation. Some studies indicate the independent role of muscle mass, volume, and density in the severity of the disease [40,41], so it could be interesting to check if obese but physically active people enjoy the same benefits of regular PA as slim ones.

Just before the COVID-19 pandemic, information on the impact of exercise on the human immune defense was published [42]. Unfortunately, the general impact of PA on infectious disease morbidity and severity was not analyzed as a pure variable but in the context of body composition (reduction of excess body mass) up to now. Our study explored and indicated the connection between PA and the severity of SARS-CoV-2 infection not related to weight loss through exercise. This information seems important not only for individuals but also for health authorities regarding healthcare direction. Although the potential biological mechanism of how PA affects our immunity at the system or cell levels, or how it modulates the release of different substances is explained in a comprehensive review by Jakobsson et al. [43]; our study complements this knowledge with clinical trial results.

## 5. Limitations

1. IPAQ-PL is dedicated to people aged 15–69. However, for the purpose of the EUPASS (European Physical Activity Surveillance System), residents who had just turned 18 were recruited [28].
2. Respondents were asked to analyze their “usual PA before COVID-19” instead of the recommended “describe your PA during the last 7 days.” In our opinion, the disease with mild symptoms/signs probably started a few days before hospitalization, and PA may have been limited. Therefore, “the last 7 days” may not adequately describe the individual’s usual status. Although the use of “the last 7 days recall” is recommended, both formulae are available [26].
3. Only patients who were not critically ill during admission were surveyed, which depends on the hospital profile and obvious reasons (patient condition).
4. The analysis of the PA during the COVID period (but not lockdown) cannot be sufficient to properly describe the long-term relationship between analyzed variables in general.
5. The correlation for CRP and PCT and physical activity level was not found as statistically significant.

## 6. Conclusions

Physical activity importantly modified the course of COVID-19 in a hospitalized population. Regular physical activity before becoming sick gives hospitalized patients about a 60% higher chance of recovery. A higher level of daily PA is associated with a milder course of SARS-CoV-2 infection.

## Figures and Tables

**Table 1:** The IPAQ level and its correlation with recovery or unfavorable hospitalization outcomes (death or transfer to ICU) and need for oxygen therapy.

Variable	IPAQ Level			Chi^2^	p-Level
	0	1	2		
**Recovery** **(N = 117)** **Death or transfer to ICU** **(N = 5 + 9)**	49	40	28		
	(37.40%)	(30.53%)	(21.37%)		
				7.15	0.03
	11	1	2		
	(8.40%)	(0.76%)	(1.53%)		
***Does the patient need oxygen therapy?***	***No***			6.78	0.03
	***(N = 48)***				
	19	12	17		
	(14.50%)	(9.16%)	(12.98%)		
	***Yes***				
	***(N = 83)***				
	***41***	***29***	***13***		
	(31.30%)	(22.14%)	(9.92%)		

Note: IPAQ—International Physical Activity Questionnaire; and ICU—Intensive Care Unit.

## Data Availability

Data (individual participant data-row data and statistical analysis, informed consent form, SAP, and study protocol) will be available after publication with no limit to all interested parties on ClinicalTrials and on https://ppm.umw.edu.pl/info/researchdata/UMW9cf7247169fc4d2b8dad0921ad2207fb/.

## Supplementary Materials

The following supporting information can be downloaded at: https://www.mdpi.com/article/10.3390/jcm12124046/s1, Figure S1: Flow diagram; Table S1: Chronic diseases in patients hospitalized due to COVID-19; Table S2: Chronic medications used by patients hospitalized due to COVID-19; Table S3: Laboratory results on admission to the hospital.
