# Supplementary material for: Physical Activity Modifies the Severity of COVID-19 in Hospitalized Patients—Observational Study
Source: J Clin Med. 2023 Jun 14;12(12):4046. doi: 10.3390/jcm12124046 (PMC10299429; doi:10.3390/jcm12124046)
Supplement: Supplementary file 1 [file jcm-12-04046-s001.zip › jcm-2424226-supplementary.pdf]

Supplementary

Figure S1. Flow Diagram.

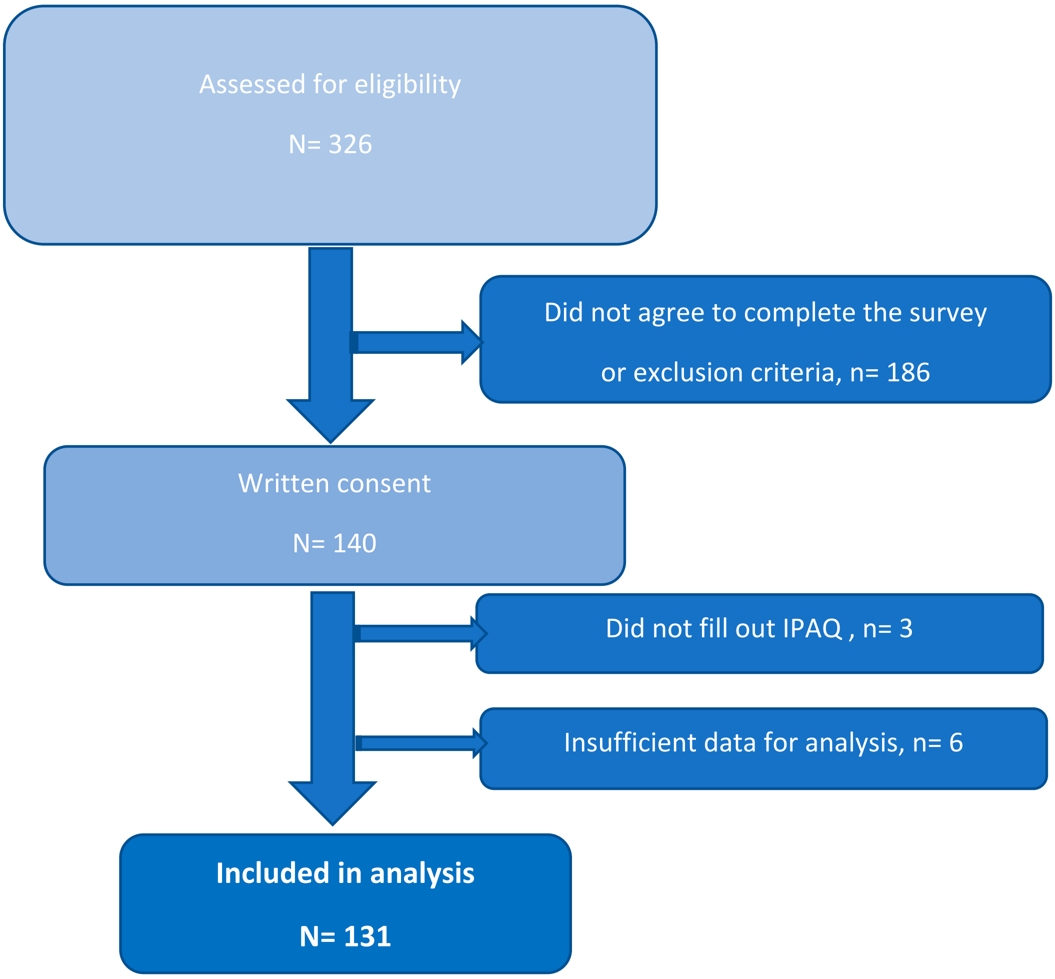

IPAQ—International Physical Activity Questionnaire

Table S1. Chronic diseases in patients hospitalized due to COVID-19.

VARIABLE N=131	OCCURRENCE	%	VARIABLE N=131	OCCURRENCE	%
CHRONIC CAD	25	19·08	OSTEOARTHRITIS	13	9·92
HF	20	15·27	INFLAMMATORY DISEASES OF THE JOINTS	5	3·82
PE	2	1·53	HISTORY OF LIMB INJURIES*	2	1·53
DVT	1	0·76	HISTORY OF CHEST INJURIES**	1	0·76
ARTERIAL THROMBOSIS	2	1·53	COPD	9	6·87
STROKE	17	12·98	BRONCHIAL ASTHMA	7	5·34
PAD	5	3·82	SEASONAL ALLERGY	1	0·76
ATHEROSCLEROSIS IN A DIFFERENT LOCATION	21	16·03	OTHER CHRONIC RESPIRATORY SYSTEM DISEASE	3	2·29
DIABETES	31	23·66	PHARMACOLOGICALLY STIMULATED IMMUNOSUPPRESSION FROM OTHER CHRONIC CONDITIONS	5	3·82

CAD—coronary artery disease; HF—heart failure; PE—pulmonary embolism; DVT—deep vein thrombosis; ALI—acute limb ischemia; PAD—peripheral artery disease;* only if affects joint mobility; ** only if limiting chest mobility; COPD—chronic obstructive pulmonary disease

Table S2. Chronic medications used by patients hospitalized due to COVID-19.

TYPE OF MEDICATION N=131	OCCURRENCE	%	TYPE OF MEDICATION N=131	OCCURRENCE	%

IMMUNOSUPPRESSIVE	11	8·40	ANTIARRHYTHMIC	27	20·61
STATINS	42	32·06	OTHER CARDIAC DRUGS	14	10·69
FIBRATES	3	2·29	RESPIRATORY SYSTEM	14	10·69
OTHER DRUGS USED IN LIPID DISORDERS TREATMENT	3	2·29	DRUGS FOR DISEASES OF THE GASTROINTESTINAL TRACT	42	32·06
ANTIHYPERTENSIVE	83	63·36	NSAIDs	3	2·29
ANTICOAGULANTS	41	31·30	HYPOGLYCEMIC DRUGS	30	22·90
ANTIPLATELET	27	20·61	OTHER DRUGS NOT INCLUDED IN THE EARLIER LIST	66	50·38

NSAIDs—nonsteroidal anti-inflammatory drugs

Table S3. Laboratory results on admission to the hospital.

**Variable**	**Valid N**	**Mean SR**	**SR-95% CI**	**SR+95% CI**	**Median**	**Min.**	**Max.**	**SD**
Hb *[g/dL]*	131	12·82	12·51	13·14	12·90	7·90	17·40	1·83
RBC *[million/µL]*	131	4·25	4·15	4·35	4·26	2·66	5·41	0·59
Ht *[%]*	131	38·41	37·57	39·25	38·30	25·10	48·20	4·86
PLT *[thousand/µL]*	131	226·73	210·49	242·98	209·00	24·00	544·00	93·98
WBC *[thousand/µL]*	131	6·50	5·97	7·03	6·10	0·95	17·93	3·04
LYMP *[thousand/µL]*	119	2·12	0·45	3·78	1·19	0·20	101·00	9·16

[%]		23·62	21·13	26·11	21·20	2·70	101·00	13·72
Glucose *[mg%]* *[mmol/L]*	127	116·20 7·18	107·41 5·63	125·00 8·74	99·00 5·49	50·00 2·78	358·00 101·00	50·07 8·84
Creatinine *[mg/dL]*	131	1·04	0·97	1·12	0·96	0·54	3·69	0·42
ALT *[U/L]*	131	41·41	33·08	49·74	25·00	5·60	376·00	48·19
AST *[U/L]*	131	49·80	39·00	60·59	35·00	12·00	575·00	62·46
LDH *[U/L]*	116	283·81	261·76	305·86	246·00	93·00	750·00	119·88
D-dimer *[µg/mL]*	125	2·37	1·40	3·33	0·94	0·22	49·66	5·45
PCT *[ng/mL]*	129	0·30	0·11	0·50	0·06	0·00	9·28	1·12
CRP *[mg/L]*	131	57·71	46·29	69·13	32·88	1·03	376·92	66·08
Albumin *[g/dL]*	86	3·49	3·39	3·59	3·50	2·30	4·70	0·46
Ferritin *[ng/mL]*	107	751·96	579·50	924·41	500·00	11·10	5342·00	899·77
TnT *[pg/mL]*	55	24·04	12·67	35·41	10·80	1·90	215·80	42·05
TCL *[mg/dL]*	56	156.16	142·97	169·35	150·50	44·00	359·00	49·25
LDL *[mg/dL]*	56	89.32	79·73	98·91	88·50	4·00	179·00	35·82
HDL *[mg/dL]*	56	37·50	33·59	41·41	34·50	11·00	88·00	14·62
TG *[mg/dL]*	56	158·25	105·81	210·69	124·00	43·00	1510·00	195·82

N—the number of individuals with laboratory data available; SD—standard deviation; Hb—hemoglobin; RBC—red blood cells; HT—hematocrit; PLT—platelets; WBC—white blood cells; LYMP—lymphocytes; ALT—alanine aminotransferase; AST—aspartate aminotransferase; LDH—lactate dehydrogenase; PCT—procalcitonin; CRP—C-reactive protein; TnT—troponin T; TCL—total cholesterol level; LDL—low-density lipoprotein; HDL—high-density lipoprotein; TG—triglycerides
